# Membranes Matter: Preventing Ammonia Crossover during Electrochemical Ammonia Synthesis

**DOI:** 10.1021/acsaem.3c02461

**Published:** 2024-01-08

**Authors:** Logan M. Wilder, Keenan Wyatt, Christopher A. Skangos, W. Ellis Klein, Makenzie R. Parimuha, Jaclyn L. Katsirubas, James L. Young, Elisa M. Miller

**Affiliations:** † Chemistry and Nanoscience Center, National Renewable Energy Laboratory, 15013 Denver W Pkwy, Golden, Colorado 80401, United States; ‡ Materials Science and Engineering Program, University of Colorado Boulder, Boulder, Colorado 80309, United States; § Department of Chemistry, University of Colorado Boulder, Boulder, Colorado 80309, United States

**Keywords:** electrochemical nitrogen reduction, electrochemical nitrate reduction, membrane, Nafion, crossover, ammonia, H-cell, gas diffusion electrode

## Abstract

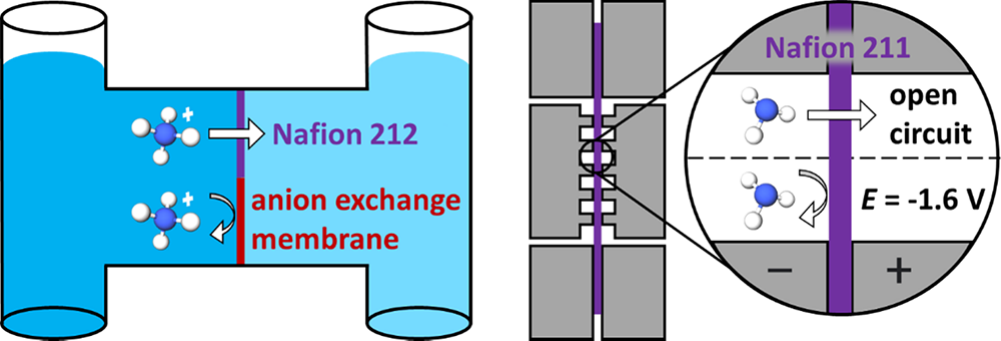

The electrochemical nitrogen and nitrate reduction reactions (E-NRR and E-NO_3_RR) promise to provide decentralized and fossil-fuel-free ammonia synthesis, and as a result, E-NRR and E-NO_3_RR research has surged in recent years. Membrane NH_3_/NH_4_^+^ crossover during E-NRR and E-NO_3_RR decreases Faradaic efficiency and thus the overall yield. During catalyst evaluation, such unaccounted-for crossover results in measurement error. Herein, several commercially available membranes were screened and evaluated for use in ammonia-generating electrolyzers. NH_3_/NH_4_^+^ crossover of the commonly used cation-exchange membrane (CEM) Nafion 212 was measured in an H-cell architecture and found to be significant. Interestingly, some anion exchange membranes (AEMs) show negligible NH_4_^+^ crossover, addressing the problem of measurement error due to NH_4_^+^ crossover. Further investigation of select membranes in a zero-gap gas diffusion electrode (GDE)-cell determines that most membranes show significant NH_3_ crossover when the cell is in an open circuit. However, uptake and crossover of NH_3_ are mitigated when −1.6 V is applied across the GDE-cell. The results of this study present AEMs as a useful alternative to CEMs for H-cell E-NRR and E-NO_3_RR electrolyzer studies and present critical insight into membrane crossover in zero-gap GDE-cell E-NRR and E-NO_3_RR electrolyzers.

## 1 Introduction

Ammonia is a critical chemical commodity in the agriculture sector and an emerging C-free fuel.^1^ Ammonia is synthesized on an industrial scale using the Haber–Bosch process, which uses elevated temperature and pressure to dissociate the strong N≡N bond.^2^ The energy input to reach elevated temperature and pressure for this process typically originates from fossil fuel inputs,^3^ and consumes up to 2% of global energy.^4^ In contrast, electrochemical ammonia synthesis using the nitrogen and nitrate reduction reactions (E-NRR and E-NO_3_RR) can be fossil-fuel-free, decentralized, and accomplished under benign conditions.^5,6^ Unsurprisingly, E-NRR and E-NO_3_RR research has surged in recent years.^2,7,8^ However, several factors complicate the study of these reactions. One such challenge is to design electrolyzers for catalyst testing to retain generated NH_3_/NH_4_^+^ while excluding contamination.^9^ A major route for NH_3_/NH_4_^+^ loss during electrochemical synthesis is membrane crossover in two-compartment cells. During catalyst evaluation experiments, such crossover results in measurement error. Herein, several commercially available membranes were screened and evaluated for use in ammonia-generating electrolyzers.

While several helpful protocols for E-NRR catalyst testing have been published,^6,10,11^ the design of E-NRR experimentation methods continues to develop.^9^ A critical challenge in E-NRR catalyst development is the high level of chemical noise (background NH_3_/NH_4_^+^) relative to the chemical signal (generated NH_3_/NH_4_^+^) in a typical E-NRR experiment, particularly in an aqueous-based electrolyte. Background NH_3_/NH_4_^+^ contamination of experimental setups can produce inflated measures of catalyst activity, and thus rigorous and expensive control experiments are required to ensure measured NH_3_/NH_4_^+^ is the result of electrocatalysis. Meanwhile, the Faradaic efficiencies of most reported E-NRR catalysts are low in aqueous-based E-NRR, often below 20%,^12^ and in benchtop-scale experiments, this results in low amounts of NH_3_/NH_4_^+^ generated relative to background levels. Several factors are responsible for the typically low Faradaic efficiency of E-NRR catalysts including the difficulty of breaking/weakening the strong N≡N bond, low solubility of N_2_ in many electrolytes, and the competing hydrogen evolution reaction.^2^ As such, retaining NH_3_/NH_4_^+^ produced electrochemically is critical to the success of the E-NRR electrolyzers.

Similar to E-NRR research, E-NO_3_RR catalyst testing methods continue to develop.^13^ The Faradaic efficiency of E-NO_3_RR catalysts is typically much higher than E-NRR catalysts in aqueous-based electrolytes, routinely reaching >80%.^14,15^ In light of this, generating sufficient NH_3_/NH_4_^+^ to significantly outcompete background contamination is easier in the E-NO_3_RR than in E-NRR experiments. However, in both E-NRR and E-NO_3_RR experiments, catalyst activity and Faradaic efficiency are assessed from ex situ quantification of NH_3_/NH_4_^+^ produced, and loss of NH_3_/NH_4_^+^ in both reduction reactions results in measurement error, which leads to underreporting of catalyst activity and Faradaic efficiency.

**Scheme 1:**
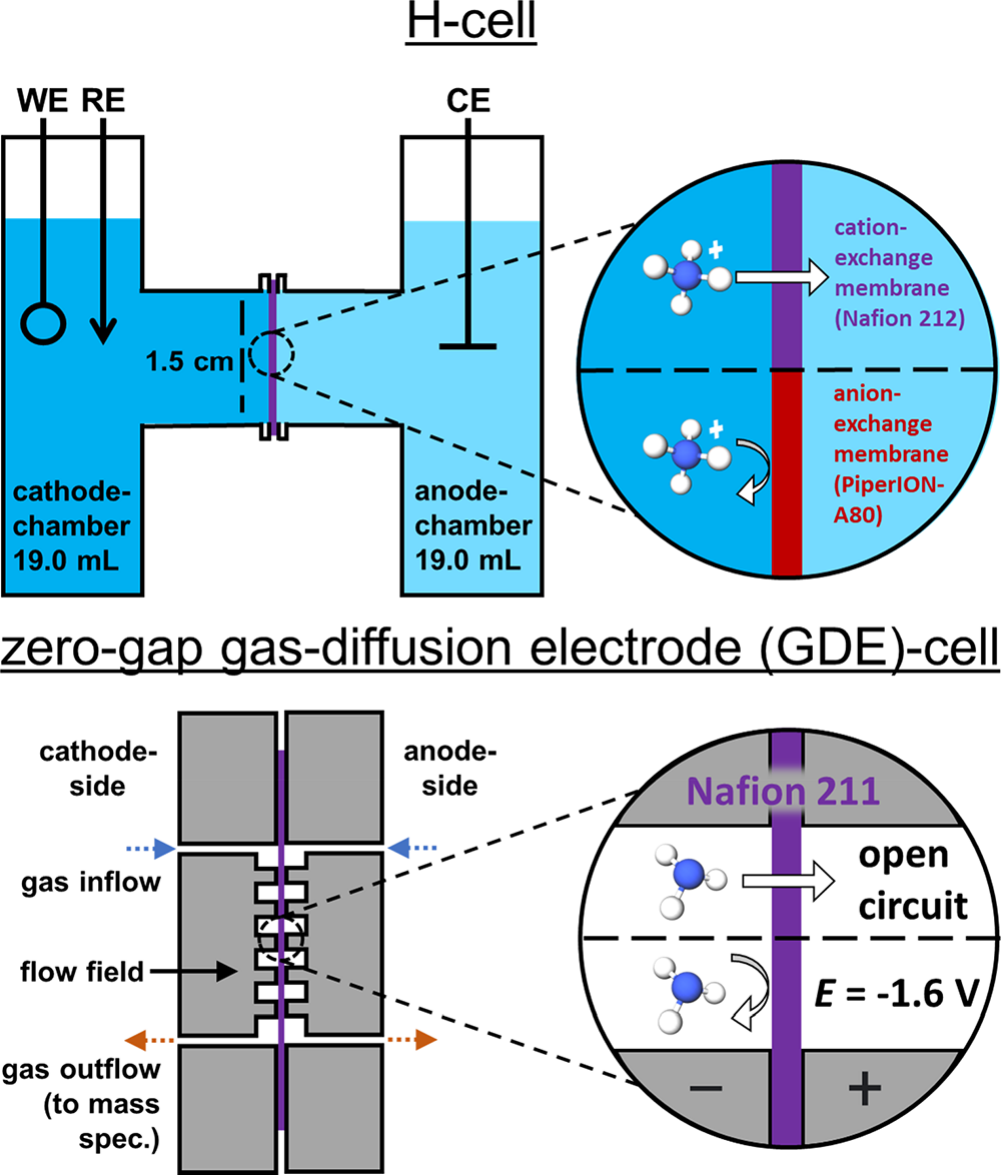
Top: H-Cell Schematic (WE = Working Electrode, RE = Reference Electrode, CE = Counter Electrode) and Illustrated Concept of NH_4_^+^ Crossover Limitation by Membrane Selection; Bottom: Zero-Gap Gas Diffusion Electrode (GDE)-Cell Schematic and Illustrated Concept of NH_3_ Crossover Control by Applied Voltage

NH_3_-generating electrolyzer catalyst testing studies frequently employ two-compartment electrochemical cells to prevent loss of NH_3_/NH_4_^+^ due to oxidation at the anode.^9,16^Scheme 1 shows two commonly used two-compartment electrochemical cells including the H-cell and the zero-gap gas diffusion electrode (GDE)-cell.^9^ H-cells are simple to implement, are generally limited to liquid-phase electrochemistry, and are widely used for E-NRR and E-NO_3_RR experiments. In contrast, GDE-based cells enable electrochemistry at a phase boundary of reactant gas, electrolyte (liquid or solid), and solid electrocatalyst. GDE-cell architecture is likely advantageous for E-NRR electrolyzers in comparison with H-cell architecture as it greatly lowers the distance required for diffusion of the sparsely soluble N_2_ from the gas phase to the catalyst active site.^17^

Two-compartment NH_3_-generating electrolyzers typically use an ion-conductive membrane as a separator between the cathode and anode chambers of the cell. It is critical to select a membrane that prevents NH_3_/NH_4_^+^ crossover and does not uptake or release significant amounts of NH_3_/NH_4_^+^. The most used membrane in E-NRR and E-NO_3_RR experiments is Nafion, a cation exchange membrane (CEM).^18,19^ Despite the widespread use of Nafion membranes, there is disagreement regarding the ability of Nafion to prevent NH_3_/NH_4_^+^ crossover.

Previous studies have assessed the usability of Nafion membranes in ammonia-generating electrolyzers.^6,18,20,21^ Relevant to this work, these studies assessed the ability of Nafion membranes to prevent NH_3_ or NH_4_^+^ crossover in H-cell architecture. Andersen et al.^6^ reported a Nafion 117 (183 μm thick)^22^ crossover experiment in pH 13.0 (0.1 M KOH) lasting 1 h, and the reported NH_3_ crossover was 5.5%. Andersen et al. also demonstrated that Nafion membranes can uptake and release NH_3_, a potential source of measurement error. Cai et al.^20^ and Ren et al.^21^ reported testing of Nafion 211, and notably to this work, qualitatively different levels of NH_4_^+^ crossover and differing conclusions on the utility of Nafion 211 in E-NRR test cells were reported. The testing procedures of these studies were similar, although not identical, both testing in pH 1.0 (0.1 M HCl) electrolyte for 2 h, and the tested NH_4_^+^ crossover of Nafion 211 ranged from only 1.0% (Cai et al.) to 38.5% (Ren et al.) between the two studies. This clear difference warrants additional study to determine the usability of Nafion membranes in E-NRR experiments.

This work presents four key points to understand and address challenges related to membrane NH_3_/NH_4_^+^ uptake and crossover in NH_3_-generating electrolyzers. Scheme 1 shows the electrolyzer architectures tested in this work, including the H-cell and GDE-cell. First, the commonly used cation exchange membrane (CEM) Nafion (specifically Nafion 212) is shown to be limited in usefulness for H-cell electrolyzers due to high measured crossover of NH_4_^+^, which would result in measurement error during a catalyst testing experiment. Second, the testing of several alternative membranes to Nafion 212 for H-cells is presented, and the anion exchange membrane (AEM) PiperION-A80 is demonstrated to show favorable properties including negligible NH_4_^+^ crossover in acidic and neutral electrolytes and negligible release of NH_4_^+^. Third, zero-gap GDE-cells show membrane NH_3_ uptake and crossover when in an open circuit, including the commonly used Nafion 211 and 212 membranes. Fourth, it is shown that an applied voltage of −1.6 V across a GDE-cell mitigates NH_3_ uptake and crossover in Nafion 211.

## 2 Results and Discussion

### 2.1 Testing Nafion 212 for NH_4_^+^ Crossover in H-Cell Architecture

Several commercially available membranes are tested to determine the NH_3_/NH_4_^+^ membrane crossover in H-cell experiments. An illustration displaying the H-cell used is shown in Scheme 1. In H-cell experiments, testing parameters such as cell dimensions, electrolyte, convection, and electrode configuration are selected to closely match typical E-NRR and E-NO_3_RR testing conditions^9,19^ and are described in detail in the Experimental Section. As shown in Scheme 1, the working electrode (WE) and reference electrode (RE) are placed in the cathode-chamber, and the counter electrode (CE) is placed in the anode-chamber. Membranes are tested for 6 h, and the cathode-chamber and anode-chamber electrolytes are sampled in 2 h increments. The concentration of NH_3_/NH_4_^+^ is evaluated using the indophenol test. Unless otherwise specified, the cathode-chamber is purged with Ar during the crossover experiments reported in this study. In this section, when the protonated or deprotonated species of the conjugate base/acid pair NH_3_/NH_4_^+^ (p*K*_a_ = 9.2)^23^ dominates equilibrium (i.e., is ≥99.9%), only the dominant species will be refereed to. All membranes are measured in triplicate (three membranes tested in three identical H-cells on the same day).

**Figure 1:**
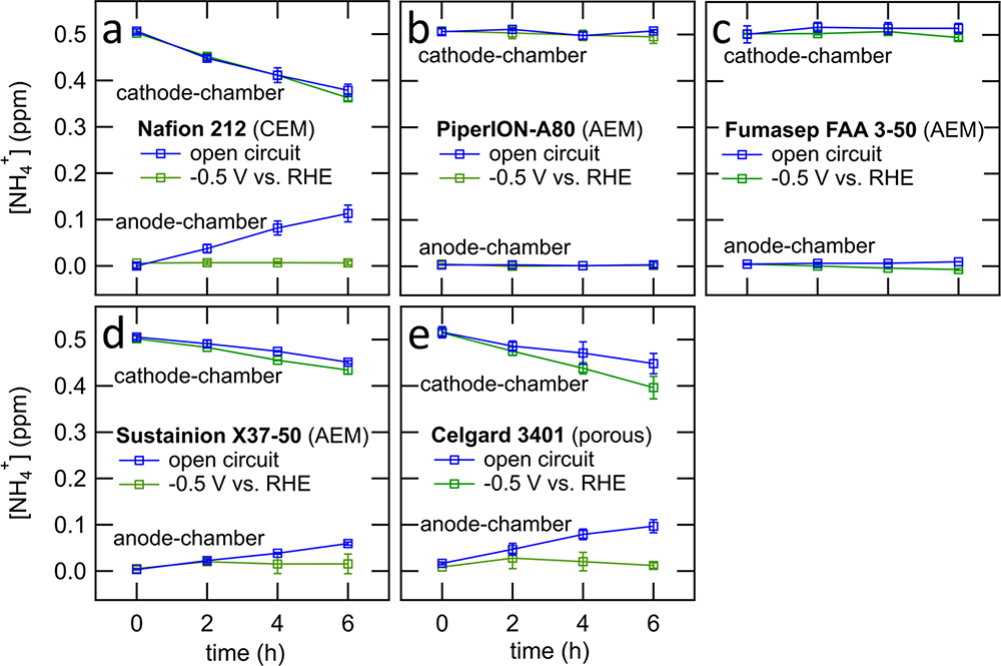
Measurement of NH_4_^+^ membrane crossover of various membranes in H-cell with membrane submerged in the electrolyte in an open circuit (blue traces) or with −0.5 V vs reversible hydrogen electrode (RHE) applied to the working electrode in the cathode-chamber of the H-cell in three-electrode configuration (green traces). The concentration of NH_4_^+^ in the cathode chamber at *t* = 0 is 0.50 ppm. The relative positions of the working electrode (WE), reference electrode (RE), and counter electrode (CE) are shown in Scheme 1. The membranes are (a) Nafion 212, (b) PiperION-A80, (c) Fumasep FAA 3–50, (d) Sustainion X37–50, and (e) Celgard 3401.

The CEM Nafion, commonly used in E-NRR and E-NO_3_RR experiments,^9,13^ is tested first. The polymer which comprises Nafion membranes contains a polytetrafluoroethylene backbone with randomly distributed perfluoroether side chains terminated with sulfonic acid groups.^24^ The specific Nafion membrane version tested in H-cell experiments is Nafion 212, which is similar in thickness (∼50 μm) to other membranes tested in H-cell experiments and frequently used NH_3_-generating electrolyzer experiments.^25−27^ A table listing the physical properties of Nafion 212 and other membranes tested in this work is provided in the Supporting Information. Figure 1 shows the results of NH_4_^+^ crossover experiments in an H-cell in an acidic electrolyte. The electrolyte in the anode-chamber is 0.1 M HCl, and the electrolyte in the cathode-chamber is 0.50 ppm of NH_4_^+^ in 0.1 M HCl. As shown in Figure 1a (blue trace), Nafion 212 clearly shows a high NH_4_^+^ crossover. The percentage retained NH_4_^+^ in the cathode-chamber at *t* = 6 h is 75 ± 2%. Table 1 also shows the percentage of retained NH_4_^+^ in both the cathode-chamber and the anode-chamber, which represents the total NH_4_^+^ in the cell except for any NH_4_^+^ trapped within the membrane. The total measured NH_4_^+^ in the cathode- and anode-chambers does not change significantly between *t* = 0 and 6 h, with the total measured NH_4_^+^ (cathode-chamber + anode-chamber) at *t* = 6 h being 99 ± 1%. This result suggests that while the Nafion 212 membrane shows significant NH_4_^+^ crossover it does not absorb or leach significant amounts of NH_4_^+^ over the course of the experiment. Concentration-versus-time values from the three replicates of the Nafion 212 open circuit NH_4_^+^ crossover test are shown in the Supporting Information (Table S1).

**Table 1:** Percentage of NH_3_/NH_4_^+^ Remaining in the H-Cell, Cathode-Chamber Only, or Entire Cell (Cathode-Chamber + Anode-Chamber) at *t* = 6 h vs *t* = 0 in Membrane Crossover Experiments

	open circuit^a^		–0.5 V vs RHE^a^	
	cathode-chamber only	entire cell	cathode-chamber only	entire cell
Nafion 212 (pH 1.0)	75 ± 2%	99 ± 1%	72 ± 1%	72 ± 1%
Fumasep FAA 3–50 (pH 1.0)	104 ± 4%	104 ± 4%	98 ± 2%	98 ± 2%
Sustainion X37–50 (pH 2.0)^b^	89 ± 1%	100 ± 2%	87 ± 3%	89 ± 3%
Celgard 3401 (pH 1.0)	87 ± 4%	102 ± 2%	77 ± 4%	78 ± 4%
PiperION-A80 (pH 1.0)	100 ± 1%	100 ± 1%	98 ± 4%	98 ± 4%
PiperION-A80 (pH 7.0)	96.7 ± 0.6%	99.9 ± 0.5%		
PiperION-A80 (pH 13.0)	80 ± 2%	93 ± 3%		

a At *t* = 0, the measured value of NH_3_/NH_4_^+^ concentration in the anode-chamber is below the limit of detection of the indophenol test in all experiments.

b Deviation in pH explained in Section 2.2.

To test Nafion 212 in an environment as close to typical E-NRR and E-NO_3_RR testing conditions as possible,^9^ Nafion 212 is also tested with the addition of a potential applied across the membrane, shown in Figure 1a (green trace). In this case, chronoamperometry is used (three-electrode mode) with a carbon paper working electrode and Ag/AgCl reference electrode on the cathode side and a graphite counter electrode on the anode side. The potential applied to the working electrode is −0.5 V vs reversible hydrogen electrode (RHE). The average current during the 6 h trial is −0.4 ± 0.1 mA, and the average full cell voltage (*E*_cathode_ – *E*_anode_) is −1.96 ± 0.08 V. A representative chronoamperogram and the full cell voltage versus time trace are shown in Figure S1. The average current and full cell voltage for all membranes tested in the H-cell are shown in Table S2. As is apparent from the data in Figure 1a, applying −0.5 V vs RHE to the working electrode does not significantly affect the NH_4_^+^ concentration versus time profile in the cathode chamber and thus does not significantly affect the rate of NH_4_^+^ crossover. The concentration profile in the anode chamber remains close to 0.0 ppm of NH_4_^+^, which is a result of NH_4_^+^ oxidation at the anode.^9,16^

As discussed in the Introduction section, there is disagreement in the recent literature concerning the usefulness of Nafion membranes for ammonia-generating electrolyzers. Both Cai et al.^20^ and Ren et al.^21^ reported testing Nafion 211 in similar experiments to this current work. Cai et al. reported NH_4_^+^ crossover of only 1.0%, while Ren et al. observed a significantly higher value of 38.5%. In the current study, Nafion 212 is tested, which is identical in composition to Nafion 211 but twice as thick, at ∼50 μm. Results of our current study indicate that Nafion 212 shows high NH_4_^+^ crossover in H-cell architecture, which is consistent with the results of Ren et al. and indicates that Nafion 212 and likely other Nafion membranes have limited usefulness as membranes for H-cell E-NRR and E-NO_3_RR experiments.

### 2.2 Testing Alternative Membranes for NH_4_^+^ Crossover in H-Cell Architecture

It is apparent from testing Nafion 212 that this membrane, and likely other Nafion membranes, allows significant NH_4_^+^ crossover in H-cell experiments on the time scale of hours. Retention of NH_4_^+^ is critical to NH_3_-generating electrolyzers employing acidic, neutral pH, or mildly basic electrolytes, and thus, a membrane with low NH_4_^+^ crossover is needed. Here, several additional commercially available membranes are tested for NH_4_^+^ crossover. These include several anion exchange membranes (AEMs) and the porous polypropylene (PP) membrane Celgard 3401. The electrolyte for H-cell NH_4_^+^ crossover experiments is 0.1 M HCl with the exception of the AEM Sustainion X37–50, which is tested with a mixed 0.01 M HCl and 0.09 M KCl electrolyte to maintain a pH of 2.0, the lowest pH recommended by the manufacturer (Dioxide Materials, Boca Raton, Florida).

The AEMs tested included PiperION-A80, Sustainion X37–50 (Grade RT), and Fumasep FAA 3–50. PiperION-A80 is composed of the polymer poly(aryl piperidinium) and is 80 μm thick.^28^ Sustainion X37–50 is described in US patent #9,370,773 as a styrene and vinylbenzyl-R (R = imidazolium or pyridinium) copolymer membrane and is 50 μm thick.^29^ The composition of Fumasep FAA 3–50 is not reported by the manufacturer (Fumatech, Bietigheim-Bissingen, Germany), and the thickness of this membrane is 50 μm. Characterization of NH_3_/NH_4_^+^ crossover of AEMs has not been reported to our knowledge.^30^

AEMs contain stationary stable cations, typically quaternary ammonium-displaying functional groups, such as the piperidinium functional group in PiperION-A80. Such nitrogen-containing polymers may release NH_3_/NH_4_^+^, originating from trapped NH_3_/NH_4_^+^ from either processing steps or decay of the polymer structure. Release of NH_3_/NH_4_^+^ by a membrane could be interpreted as false positive electrocatalytic NH_3_/NH_4_^+^ generation, and so the as-received AEMs were tested for bound NH_3_/NH_4_^+^. To test for the release of NH_3_/NH_4_^+^ from AEMs and other membranes, the membranes were soaked in the electrolyte to release bound NH_3_/NH_4_^+^, and the resulting soaking solutions were tested. Specifically, 3.0 × 3.0 cm pieces of all as-received membranes were soaked in 0.1 M HCl (40 mL) for 18 h and the indophenol test was performed on the resulting soak solution. It was found that PiperION-A80 does not release measurable NH_3_/NH_4_^+^, while Sustainion X37–50 releases 1.70 μg and Fumasep FAA 3–50 releases 0.95 μg. Comparatively, Nafion 212 did not release measurable NH_3_/NH_4_^+^ in this test. Testing results of other membranes in this study (as-received) are shown in Table S3 of the Supporting Information.

AEMs likely restrict NH_4_^+^ transport due to charge exclusion of the NH_4_^+^ cation. It was therefore predicted that AEMs would show low NH_4_^+^ crossover in comparison to that of the CEM Nafion 212. This prediction is confirmed for the three AEMs tested. These AEMs tested show low NH_4_^+^ crossover in comparison with Nafion 212 as shown in Figure 1b–d and Table 1. The PiperION-A80 and Fumasep FAA 3–50 membranes show negligible NH_4_^+^ crossover in both open circuit and −0.5 V vs RHE trials, while the Sustainion X37–50 membrane does display some crossover. Concentration versus time values from the three replicates of the PiperION-A80 open circuit NH_4_^+^ crossover test are shown in the Supporting Information (Table S4).

Of the AEMs, PiperION-A80 shows the best performance as a membrane for NH_3_-generating electrolyzers. Specifically, PiperION-A80 shows no measurable NH_4_^+^ crossover, and in addition, the membrane does not release measurable quantities of NH_4_^+^ in the H-cell crossover experiment or in the characterization of the as-received membrane (Table S3). As such, PiperION-A80 is a useful membrane for E-NRR and E-NO_3_RR experiments in an acidic electrolyte. It should be noted that as an AEM, PiperION-A80 will likely show higher ionic resistance in acidic electrolytes in comparison with CEMs; however, the parameter of membrane ionic resistance does not affect the results of catalyst testing experiments that are conducted in a three-electrode configuration. Moreover, as shown in Table S2, the average full cell voltage (*E*_cathode_ – *E*_anode_) for all AEM trials is ≤−2.1 V, a value that is well within the compliance voltage of the typical laboratory potentiostat. In addition to AEMs, the PP membrane Celgard 3401 is also tested for NH_4_^+^ crossover. Porous PP membranes have been recommended for E-NRR experiments because they are inexpensive, require no preconditioning, and PP does not uptake or release significant amounts of NH_3_/NH_4_^+^.^6,31^ Celgard 3401 is 25 μm thick, surfactant coated, and 41% porous, as described by the manufacturer (Celgard LLC, Charlotte, NC).

As a porous membrane, it was expected that Celgard 3401 would allow NH_4_^+^ crossover, and this is confirmed by the results shown in Figure 1e and Table 1. As shown in Table 1, Celgard 3401 displays a level of NH_4_^+^ crossover similar to that of Nafion 212. As Celgard 3401 is porous, the driving force defining the crossover rate is the diffusion of NH_4_^+^ across the open channels of the PP barrier. In a previous literature report, Andersen et al.^6^ reported evaluating Celgard 3401 in an H-cell NH_3_ crossover test with pH 13.0 (0.1 M KOH) electrolyte (1.0 h), and in this study, negligible NH_3_ crossover was measured in open circuit experiments, but NH_3_ crossover was found to increase significantly with the application of a potential across the membrane. The difference between the findings of Andersen et al.^6^ and the current study is likely due to the differences in pH, testing time, and the use of forced convection by stirring in the experiments reported here. The high level of NH_4_^+^ crossover of Celgard 3401 limits the usefulness of this membrane for E-NRR and E-NO_3_RR experiments.

### 2.3 Testing PiperION-A80 in Neutral pH and Basic Electrolytes

**Figure 2:**
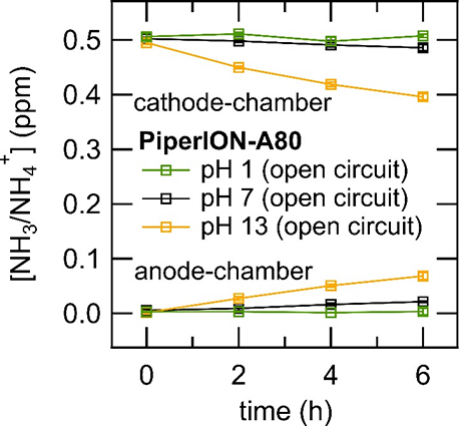
Measurement of NH_3_/NH_4_^+^ crossover of PiperION-A80 in the H-cell in an open circuit with the membrane submerged in various electrolytes. The concentration of NH_3_/NH_4_^+^ in the cathode chamber at *t* = 0 is 0.50 ppm of NH_4_^+^. The pH 1.0 electrolyte is 0.1 M HCl, the pH 7.0 electrolyte is 0.1 M potassium phosphate buffer, and the pH 13.0 electrolyte is 0.1 M KOH. In the pH 1 trial, the cathode-chamber is purged with Ar throughout the experiment, while in the case of pH 7 and 13 trials, Ar purging is not used to prevent loss of NH_3_ to the atmosphere.

Electrolytes for E-NRR and E-NO_3_RR range from acidic to basic.^10,14^ Therefore, here, PiperION-A80 is also tested in neutral pH and basic electrolytes. PiperION-A80 NH_3_/NH_4_^+^ crossover testing is repeated with pH 7.0 (0.1 M potassium phosphate buffer) and pH 13.0 (0.1 M KOH) electrolytes in an open circuit. In the case of these neutral pH and basic experiments, Ar purging of the electrolyte is not used to prevent the loss of NH_3_ to the atmosphere. The results are shown in Figure 2 and Table 1. Increasing the electrolyte pH from 1.0 to 7.0 shows a small increase in NH_3_/NH_4_^+^ crossover, while a further increase to pH 13.0 is accompanied by a much larger increase in NH_3_ crossover. As previously stated, the p*K*_a_ of NH_3_/NH_4_^+^ is 9.2, and thus a likely contributing factor to the increase in NH_3_/NH_4_^+^ crossover with increased pH is the greater equilibrium proportion of NH_3_ to NH_4_^+^. AEMs rely primarily on charge exclusion to prevent the transport of cations, and so it is likely that the charge exclusion mechanism that prevents NH_4_^+^ crossover does not prevent neutral NH_3_ from crossing the membrane. While the electrolyte pH in these trials is within the recommended pH range reported by the manufacturer of PierION-A80 (pH = 1.0–14.0, Versogen, Newark, Delaware), it is also possible that pH-mediated changes in the morphology or chemistry of the PiperION-A80 polymer structure contribute to changes in NH_3_/NH_4_^+^ crossover. Additionally, in the case of pH 13.0 electrolyte, the total NH_3_ (cathode-chamber + anode-chamber) at t = 6 h is 93 ± 3% of the *t* = 0 value. In this case, it is possible that the membrane uptake of NH_3_ is responsible for this decrease. Finally, while the pH 7.0 and pH 13.0 electrolytes were stirred, the absence of sparging in these trials resulted in decreased forced convection in relation to the pH 1.0 experiment.

It is apparent from the results of crossover testing in neutral pH and basic electrolytes that PiperION-A80 is useful as a membrane for NH_3_-generating electrolyzers with electrolyte pH ranging from pH = 1.0–7.0. In systems employing electrolytes in the range of pH above pH = 7.0, control experiments to determine the NH_3_/NH_4_^+^ crossover rate in a specific electrolyzer system should be carried out to determine if the rate of crossover is acceptable.

### 2.4 Gas-Phase NH_3_ Membrane Crossover in the Zero-Gap Gas Diffusion Electrode (GDE)-Cell

Gas diffusion electrode (GDE)-based cell architectures provide high availability of gas-phase N_2_ at the electrode surface relative to the solubility-limited N_2_ concentration of liquid electrolytes, and thus, there is significant interest in GDE-cell E-NRR electrolyzers.^17^ Here, several commercially available membranes are tested to determine NH_3_ membrane crossover in a zero-gap GDE-cell (Scheme 1).

**Figure 3:**
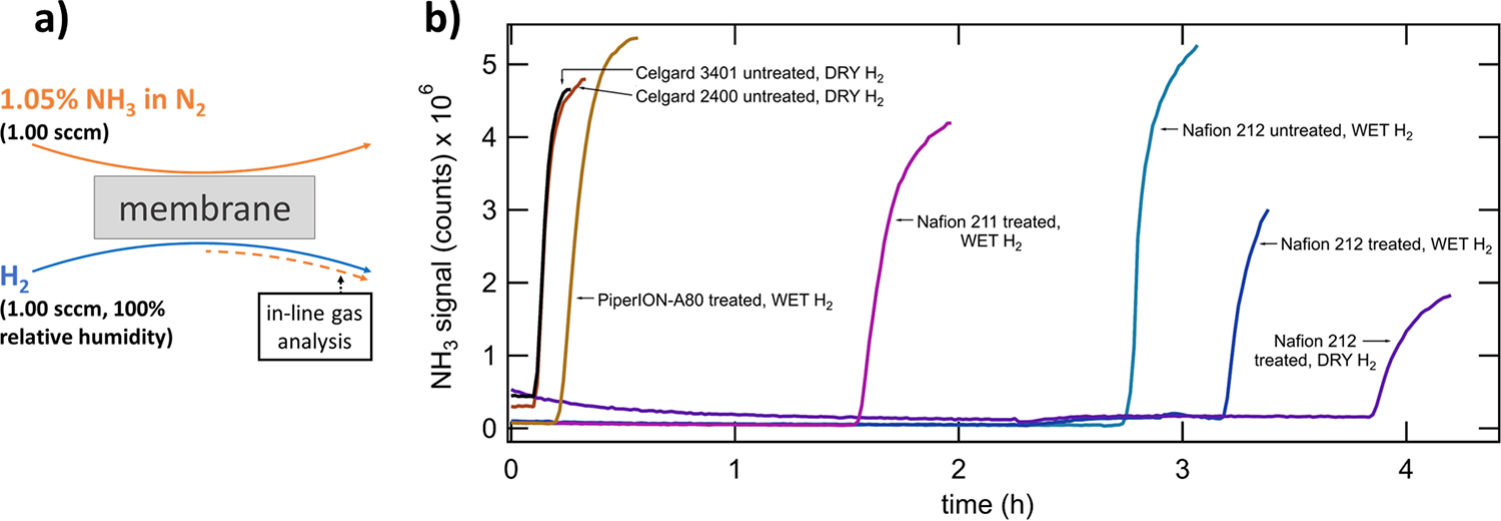
(a) Schematic of gas-phase NH_3_ crossover monitoring. (b) In-line measurement of NH_3_ crossover onset at the GDE-cell anode outlet for various membranes. After an initial 5.0 min of N_2_ feed to the cathode-inlet, the feed is switched to 1.05% NH_3_ in N_2_ for the remaining duration of each test. The labels “WET H_2_” and “DRY H_2_” refer to humidified or nonhumidified H_2_ supplied to the anode-side of the GDE-cell.

The GDE-cell crossover testing parameters are described in the Experimental Section and are summarized as follows. In this section, the side of the cell supplied with NH_3_ is referred to as the “cathode side”, and unless otherwise specified, the crossover experiments are performed in an open circuit. The initial cathode-side gas feed at *t* = 0 is N_2_, while the anode-side is exposed to a continuous flow of H_2_ (dry or humidified). At 5.0 min, the cathode-side gas feed is switched to 1.05% NH_3_ in N_2_. The anode-side cell effluent is continuously sampled for analysis by time-of-flight mass spectrometry (TOF-MS) as displayed in the illustration in Figure 3a. Note that slight differences in the initial baseline NH_3_ signal result from differences in the time allowed between tests for cell purging and TOF-MS chamber evacuation.

Data showing the onset of NH_3_ membrane crossover in the GDE-cell are shown in Figure 3b. The onset of crossover is indicated by a sharp increase in the intensity of the NH_3_ signal in each trace. The “treated” label in the figure refers to membranes that are prepared as described in the Experimental Section, while the “untreated” label refers to membranes that are tested as-received from the manufacturer with no pretreatment. The “treated” membranes are water-saturated prior to the test, while the “untreated” membranes are not. The “DRY H_2_” label refers to dry H_2_ supplied to the anode side, while the “WET H_2_” label refers to humidified H_2_ supplied to the anode side. Two Celgard membranes, Celgard 2400 and 3401, are tested, and both membranes display NH_3_ crossover onset within minutes of the introduction of NH_3_, as could be expected from this class of highly porous PP membranes. A series of Nafion membranes are tested, including Nafion 211 and 212, with the Nafion 212 tested in variations of “treated” and “untreated” and with dry or humidified H_2_. The treated Nafion 211 and 212 tested under humidified H_2_ conditions show NH_3_ crossover onsets of 1.53 and 3.18 h, corresponding to the difference in thickness (25 and 50 μm, respectively) of the compositionally identical membranes. The treated Nafion 212 tested with dry H_2_ shows a longer NH_3_ crossover onset of 3.83 h. This difference may correspond to the dry H_2_ removing more water from the membrane during the test than the humidified H_2_ condition. Untreated Nafion 212 tested with humidified H_2_ shows the earliest crossover onset of the Nafion 212 tests, 2.73 h. The apparent NH_3_ crossover of all Nafion membranes in an open circuit is a predictable result as perfluorosulfonic acid membranes have been previously shown to display high NH_3_ permeability.^32^

In addition to testing Nafion and Celgard membranes, Figure 3b shows crossover testing of PiperION-A80, which displayed low NH_4_^+^ crossover in H-cell testing (previous section). The membrane is treated as described in the Experimental Section and tested under humidified H_2_ conditions. The PiperION-A80 membrane shows the fastest onset of the NH_3_ crossover among all nonporous membranes.

The obtained zero-gap GDE-cell membrane crossover results clearly show that when the cell is in an open circuit all tested membranes show NH_3_ crossover. Thus, NH_3_-generating electrolyzer operating procedures with discontinuous operation, such as those possible when energy is supplied from variable renewable sources, must consider the likelihood of NH_3_ crossover during periods of the cell in an open circuit.

### 2.5 NH_3_ Crossover during GDE-Cell Operation

Operation of GDE-cells includes an externally applied voltage, and so here, Nafion 211, which is commonly used in GDE-cell devices, is tested with an applied voltage of −1.6 V across the cell. Initial testing demonstrated notable membrane NH_3_ uptake and release behavior in response to applied voltage, and so in this section, NH_3_ uptake is measured (instead of crossover as in the previous section) by monitoring NH_3_ in the cathode-side effluent. Parameters for the GDE-cell NH_3_ crossover test with applied voltage are described in the Experimental Section and are summarized here.

**Figure 4:**
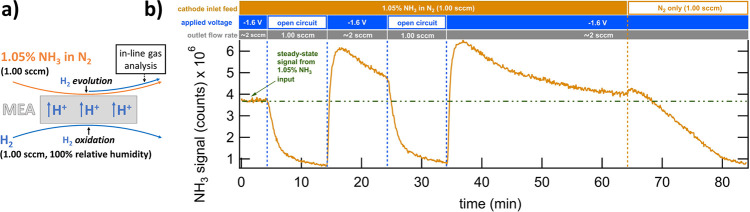
(a) Schematic of membrane NH_3_ uptake/release monitoring. (b) NH_3_ signal vs time at GDE-cell cathode outlet with a cell inlet feed of 1.05% NH_3_ while alternating the cell between open circuit and −1.6 V with a steady-state current of −32 ± 1 mA/cm^2^ (geometric area). The symmetric cell employs a GDE with a Pt/C catalyst on each side of the Nafion 211 membrane.

Figure 4a shows two key differences between the NH_3_ uptake test setup and the test setup for crossover (Figure 3a). First, the in-line gas analysis sampling occurs on the cathode outlet, and second, a symmetric membrane-electrode assembly (MEA) is used that consists of a Nafion 211 membrane sandwiched by GDEs on either side. The addition of the GDEs is necessary to pass current through the MEA, and it should be noted that it is possible that the addition of the GDEs could influence the transport of NH_3_ through the cell, for instance, by acting as a barrier between the flow field and the membrane.^34^ In a control experiment, NH_3_ transfer from the cathode-side flow field to the anode-side flow field occurred ∼1 min later in a cell containing a single GDE versus a blank (no membrane, no GDE) cell, as shown in Figure S2. In cell operation, H_2_ is supplied to the anode in excess and is oxidized to generate protons (H^+^) which move across the membrane to the cathode where protons are reduced to H_2_. The oxidation of NH_3_ is a possible additional anode reaction, however, NH_3_ crossover to the anode side is likely to be negligible within the time scale of the experiment (80 min) as informed by the results of the Nafion 211 NH_3_ crossover test, which shows ∼90 min of operation of the cell in an open circuit is necessary to observe NH_3_ crossover. The test conditions are varied during the experiment, specifically the cathode-inlet gas composition (orange bar, top of Figure 4b) and the applied voltage (blue bar, top of Figure 4b). When a voltage of −1.6 V is applied across the cell, the steady-state current is −32 ± 1 mA·cm^–2^ (geometric area). The cathode outlet flow rate (gray bar, top of Figure 4b) is approximately 2 sccm when a voltage of −1.6 V is applied across the cell, and this represents a mixture of N_2_ and H_2_, as the H_2_ generation rate is approximately 1 sccm.

The Nafion 211 NH_3_ uptake measurement with and without applied voltage is shown in Figure 4b. Prior to the start of the test, the cell is operated at −1.6 V and fed with 1.05% NH_3_ on the cathode side until a steady-state NH_3_ signal is established (*t* = 0). At *t* = 4.3 min, the voltage source is turned off, leaving the cell in an open circuit. The resulting drop in the NH_3_ signal indicates NH_3_ absorption/crossover of all or nearly all of the inlet NH_3_ with the NH_3_ signal approaching zero within *∼*10 min. At this point (*t* = 14.2 min), the cell is returned to −1.6 V, immediately yielding a sharp increase in NH_3_ in the cathode-side effluent that exceeded the original steady-state value before gradually decreasing. This excess NH_3_ release at −1.6 V likely corresponds to the NH_3_ uptake that occurred during the previous segment in the open circuit. This cycle is repeated starting at *t* = 24.5 min by returning to an open circuit for 10 min and then to −1.6 V at *t* = 34.2 min. In this cycle, the NH_3_ signal is allowed 30 min to return to its original steady-state level of *t* = 0. Then, at *t* = 64.2 min, the 1.05% NH_3_ feed is switched to pure N_2_, showing the purge of residual NH_3_ from the gas supply lines and cell flow field over the course of 20 min.

Two important points are apparent from the GDE-cell testing data in Figure 4. First, when the cell is in an open circuit, most or all of the NH_3_ entering the cell cathode-side does not exit the cell cathode-side. This result indicates that in an open circuit the MEA uptakes a significant amount of NH_3_. Such a result agrees with the crossover testing of Nafion 211 (Figure 3b) because for NH_3_ crossover to occur the membrane must first uptake NH_3_. Second, the application of a voltage across the cell appears to both halt the uptake of NH_3_ by the MEA while initiating the release of stored NH_3_. The mechanism for the apparent halting of NH_3_ uptake and release of stored NH_3_ may be electrophoresis, electroosmotic drag (EOD), or a combination of these factors dependent on the speciation of the NH_3_/NH_4_^+^ conjugate base/acid pair within the membrane.^35^ As the pH may vary within the membrane, NH_3_ within the membrane could exist primarily as NH_3_ or NH_4_^+^. The NH_4_^+^ species would experience the forces of both electrophoresis and EOD while the uncharged NH_3_ species would experience only the force of EOD.

As is apparent in Figure 4b, applying a voltage across the GDE-cell, a fundamental part of cell operation, induces advantageous halting of NH_3_ membrane uptake and releases NH_3_ stored within the membrane. This finding presents an encouraging picture of the feasibility of zero-gap GDE-cell-based E-NRR electrolyzers. Moreover, it is important to note that previous studies have demonstrated that Nafion 211 and other membranes can contain NH_3_ as-received from the manufacturer or absorbed from pretreatment or test solutions.^18,36^ The results of this study indicate that such NH_3_ contamination stored within a Nafion 211 membrane would likely travel to the cathode outlet of a GDE-cell upon voltage turn-on, and this release of NH_3_ might be erroneously attributed to E-NRR activity. In this hypothetical case of employing a membrane containing contaminant NH_3_, the spike in the NH_3_ signal would likely be lower than that shown in Figure 4b, as the membrane in this study was deliberately exposed to a relatively high concentration of NH_3_ for a substantial period. It is therefore critical to conduct thorough control experiments and ensure that membranes are free of NH_3_ prior to the start of a GDE-cell E-NRR electrocatalysis test.

## 3 Conclusions

An investigation of the performance of various membranes for two-compartment cell NH_3_-generating electrolyzers is conducted. Effective strategies for limiting NH_3_/NH_4_^+^ crossover in both H-cell and zero-gap gas diffusion electrode (GDE)-cell electrolyzer architectures are presented.

In H-cell tests, the commonly used CEM Nafion, specifically Nafion 212, is shown to be readily crossed by NH_4_^+^_._ This represents a major limitation to the use of Nafion CEMs in NH_3_-generating H-cell electrolyzers. Several alternative membranes are investigated, including AEMs and PP membranes. It is found that AEMs show greatly reduced or negligible NH_4_^+^ crossover and the porous PP membrane showed high NH_4_^+^ crossover. The AEM PiperION-A80 does not release NH_3_/NH_4_^+^ into any electrolytes, and the membrane shows negligible crossover of NH_4_^+^ in acidic and neutral pH electrolytes. However, PiperION-A80 is readily crossed by NH_3_ in a basic (pH 13) electrolyte. This result highlights that the AEM PiperION-A80 is a useful membrane for NH_3_-generating H-cell electrolyzer experiments, such as catalyst testing, in acidic and neutral electrolytes.

In zero-gap GDE-cell tests, most membranes tested show significant NH_3_ crossover in an open circuit, and the crossover onset times vary from just minutes to several hours. Nafion 212 is tested with and without humidification and shows a shorter crossover onset time when humidified. In additional testing, it is demonstrated that the application of −1.6 V across the GDE-cell (generating −32 ± 1 mA·cm^–2^) mitigates NH_3_ uptake in Nafion 211. The likely mechanisms for this are the electroosmotic drag of NH_3_ and NH_4_^+^ and electrophoresis of NH_4_^+^ transporting and confining these species to the cathode side of the membrane. This result shows that voltage turn-on in a GDE-cell may be accompanied by the release of any NH_3_ present within the membrane. In the case of a membrane containing contaminant NH_3_, the release of the contaminant NH_3_ from a membrane at the beginning of an experiment could be falsely interpreted as catalytic NH_3_ generation and should be considered in the design of control experiments. Additionally, NH_3_-generating electrolyzer operating procedures with discontinuous operation, such as is possible when energy is supplied from variable renewable sources, must consider the likelihood of NH_3_ crossover during periods the cell is in an open circuit. Parameters of GDE-cell and H-cell operation (concentration, speciation of NH_3_/NH_4_^+^, batch versus flow, etc.) are quite different, so as we show here, one should not expect direct translation of the H-cell NH_4_^+^ retention experiments to the GDE-cell NH_3_ crossover onset times. This highlights the importance of performing cell-architecture-specific membrane crossover testing and electrocatalyst control experiments to understand and accurately reflect the performance of a given cell architecture.

## 4 Experimental Section

### 4.1 Chemicals and Materials

Hydrochloric acid, sodium hydroxide, potassium hydroxide, sodium citrate (tribasic), salicylic acid, potassium phosphate (dibasic), potassium phosphate (monobasic), sodium hypochlorite solution (10–15%), and sodium nitroferricyanide(III) dihydrate were purchased from Millipore Sigma (Burlington, Massachusetts). Sustainion X37–50 grade RT, PiperION-A80, Fumasep FAA 3–50, and isomolded graphite plates were purchased from The Fuel Cell Store (College Station, Texas). Nafion 211 and Nafion 212 were purchased from Fuel Cell Earth (Woburn, Massachusetts). Celgard 3401 and Celgard 2400 were purchased from Celgard (Charlotte, North Carolina). All solutions were made using deionized (DI) water (>18.0 MΩ·cm, Milli-Q Gradient System, Millipore Sigma). Custom glass H-cells were purchased from Adams & Chittenden Scientific Glass (Berkeley, California).

### 4.2 Membrane Preparation

Membranes were prepared according to manufacturer recommendations or following commonly used procedures.^24^ To prepare Nafion 211 and Nafion 212, the membranes were first immersed in aqueous 5.0% H_2_O_2_ at 90 °C for 1 h, then rinsed with DI water, then immersed in 0.5 M H_2_SO_4_ at 90 °C for 1 h, then rinsed with DI water again, and finally, the membranes were immersed in DI water at 90 °C for 1 h. To prepare Sustainion X37–50, the membrane was immersed in 1.0 M NaOH for 18 h, then rinsed with DI water, then the membrane was immersed in a solution composed of 10.0 mM HCl and 90.0 mM KCl for 1 h, and finally, the membrane was rinsed with DI water. To prepare PiperION-A80 and Fumasep FAA 3–50, these membranes were immersed in 0.1 M HCl for 18 h and then rinsed with DI water. Celgard 3410 was rinsed with DI water.

### 4.3 Membrane Crossover Testing in H-Cells

A glass H-cell was assembled with the membrane of choice. The inner diameter of the H-cell orifice was 1.50 cm. The electrode configuration is shown in Scheme 1. Electrodes in the cell included a carbon paper working electrode (1.0 cm × 2.0 cm, AvCarb MGL370, Fuel Cell Store), a Ag/AgCl reference electrode (3 M KCl, BASi Research Products, West Lafayette, Indiana), and a graphite plate counter electrode. The working and reference electrodes were placed in the cathode chamber of the H-cell, and the counter electrode was placed in the anode chamber of the H-cell. In all H-cell experiments, including experiments in an open circuit, prior to the 6 h crossover test, an electrochemical cell preconditioning step was carried out. A discussion of the necessity of the cell preconditioning step is included in the Supporting Information. In the electrochemical preconditioning step, chronoamperometry was used to hold the working electrode at −0.5 V vs RHE for 1 h. The electrolyte used was the same electrolyte that was used for the following crossover test, that is, either 0.1 M HCl, 0.1 M potassium phosphate buffer, or 0.1 M KOH. Following the 1 h preconditioning step, the assembled H-cell was rinsed with DI water three times, and then, the cathode-chamber of the H-cell was rinsed with 0.50 ppm of NH_4_^+^ in the selected electrolyte and the anode-chamber was rinsed with the selected electrolyte. Next, the cathode chamber was filled with 19.0 mL of 0.50 ppm of NH_4_^+^ in the electrolyte of interest, and the anode chamber was filled with 19.0 mL of the electrolyte of interest. Both the cathode-chamber and anode-chamber of the H-cell were stirred during the crossover experiment with magnetic stir bars (1.5 mm × 8 mm size, Teflon-coated, 750 rpm rotation rate). Ar gas was bubbled into the cathode chamber at a rate of 13.0 mL/min for the entire span of the crossover experiment when testing membranes in 0.1 M HCl (pH 1.0) or 0.01 M HCl + 0.09 M KCl (pH 2.0) electrolyte. In the case of the pH 1.0 and pH 2.0 electrolyte membrane crossover experiments, the speciation of NH_3_/NH_4_^+^ overwhelmingly favors the nonvolatile NH_4_^+^ species (>99.999%), and therefore in these experiments, the electrolyte within the H-cell was a sufficient trap to prevent loss of NH_3_ to atmosphere. No gas bubbling was employed when testing membranes with 0.1 M potassium phosphate buffer or 0.1 M KOH electrolytes to minimize the loss of NH_3_ to the atmosphere. Three identical replicate H-cells were prepared for each experiment. Aliquots of 1.00 mL were collected at times 0, 2, 4, and 6 h. Two two-channel potentiostats (Bio-Logic USA, Model SP300, Knoxville, Tennessee) were used in the chronoamperometry mode for electrochemical experiments.

### 4.4 Indophenol Test

To assess NH_3_/NH_4_^+^ concentration in aqueous samples, the indophenol test was used.^12^ First, a 1.00 mL aliquot of the solution to be tested was taken from the H-cell. Next, 1.00 mL of an aqueous solution containing 1.0 M NaOH, 0.170 M sodium citrate, and 0.362 M salicylic acid was added. Next, 0.500 mL of ∼70 mM sodium hypochlorite in water was added. Finally, 0.100 mL of 22.4 mM sodium nitroferricyanide(III) in water was added. The solution was vigorously mixed and then incubated for 2 h, and then, the absorbance at 655 nm was measured by a UV–vis spectrometer (Cary 7000, Agilent, Santa Clara, California). The indophenol test calibration curve was remeasured for each sample set measured on a given day. A sample indophenol test calibration curve is shown in Figure S3 of the Supporting Information. UV–vis spectra of NH_4_^+^ standards with concentrations 0.00–0.50 ppm are shown in Figure S4 of the Supporting Information.

### 4.5 Membrane Crossover Testing in the GDE-Cell in the Open Circuit

The gas-phase NH_3_ crossover tests were performed by using a custom test bench designed for in situ testing of GDE-based E-NRR cell architectures. The cell effluent was continuously, in-line sampled by a multiturn time-of-flight mass spectrometer (TOF-MS) (JEOL infiTOF, JEOL USA Inc., Peabody, Massachusetts). The cell hardware consisted of stainless-steel anode- and cathode-side flow fields, current collectors, and end plates with eight clamping bolts. The flow field plates had a single serpentine flow pattern over a 5 cm^2^ area. The tested membrane was placed between the flow field plates with 1 mil (25.4 μm) thick PTFE gaskets (5 cm^2^ opening) on either side of the membrane to define the active area. Membrane NH_3_ crossover measurements were conducted by supplying 1.00 sccm of 1.05% (v/v) NH_3_ (balance N_2_) calibration gas standard (Cal Gas Direct, Huntington Beach, California) to the cell cathode-side flow field while 1.00 sccm of dry or humidified H_2_ was supplied to the anode-side flow field. The cathode-side gas feed was not humidified, as the large water volume and surface area within a humidifier would act as a trap and reservoir for NH_3_ in the gas supply stream. The initial cathode-side gas feed at *t* = 0 was 1.00 sccm N_2_. At 5.0 min, the cathode-side gas feed was switched to 1.00 sccm of 1.05% NH_3_ in N_2_. The NH_3_ crossover signal was monitored for up to 4.50 h or until the onset of the NH_3_ crossover was observed. All GDE-cell experiments were conducted at an ambient room temperature of 21–23 °C.

### 4.6 Membrane Crossover Testing in the GDE-Cell during Cell Operation

Parameters for membrane crossover testing in the GDE-cell during cell operation were identical to the previous section with the exception of the following alterations. A MEA (symmetric) was tested rather than only a membrane. The MEA consisted of a Nafion 211 membrane with GDEs added to each side of the membrane in order to pass current. Each GDE (Freudenberg H23C8, The Fuel Cell Store, Bryan, Texas) contained a carbon-based gas diffusion layer with a microporous carbon layer and a catalyst layer consisting of Pt (50 wt %) supported on high-surface-area carbon.^33^ Additionally, the in-line gas analysis sampling occurred at the cathode-side gas outlet rather than the anode-side outlet in order to measure NH_3_ uptake. Also, during some stages of the GDE-cell operation test, a voltage of −1.6 V was applied across the GDE-cell, as indicated in Figure 4b. Finally, the cathode side of the GDE-cell was supplied with either 1.00 sccm 1.05% NH_3_ in N_2_ or N_2_ only during different stages of the test, as indicated in Figure 4b.

### 4.7 Gas Analysis

Gas analysis was performed by a continuous sampling of the cell effluent via 50 μm inner diameter capillary tubing (PEEKsil, Supelco 51332-U, Millipore Sigma) connected to the sample insertion interface of the time-of-flight mass spectrometer (TOF-MS).^37^ The TOF-MS was configured with a four-turn flight path, 10 eV ionization energy, 40 μA ion current, 100 °C ion chamber temperature, and 2400 V detector voltage.

## Supporting Information Available

Tables showing additional data from H-cell NH_4_^+^ crossover experiments; table showing properties of membranes used in the study; representative current and voltage traces; GDE-cell NH_3_ crossover signals with and without GDE present; representative indophenol test calibration curve and spectra; and discussion of graphite anode as a source of contaminating NH_3_/NH_4_^+^ (PDF)

## Abbreviations

E-NRR: electrochemical nitrogen reduction reaction
E-NO_3_RR: electrochemical nitrate reduction reaction
CEM: cation exchange membrane
GDE: gas diffusion electrode
AEM: anion exchange membrane
RHE: reversible hydrogen electrode
PP: polypropylene
DI: deionized
TOF-MS: time-of-flight mass spectrometry
MEA: membrane-electrode assembly
EOD: electroosmotic drag
