# Supplementary material for: Membranes Matter: Preventing Ammonia Crossover during Electrochemical Ammonia Synthesis
Source: ACS Appl Energy Mater. 2024 Jan 8;7(2):536–45. doi: 10.1021/acsaem.3c02461 (PMC10806602; doi:10.1021/acsaem.3c02461)
Supplement: Supplementary file 1 — ae3c02461_si_001.pdf [file ae3c02461_si_001.pdf]

Supporting Information

Membranes Matter: Preventing Ammonia Crossover During Electrochemical Ammonia Synthesis

Logan M. Wilder[1], Keenan Wyatt[1,2], Christopher A. Skangos[1], W. Ellis Klein[1], Makenzie R. Parimuha[1], Jaclyn L. Katsirubas[1,3], James L. Young[1], and Elisa M. Miller[1]**

[1]Chemistry and Nanoscience Center, National Renewable Energy Laboratory, 15013 Denver W Pkwy, Golden, CO 80401, USA

[2]Materials Science and Engineering Program, University of Colorado Boulder, Boulder, CO 80309, USA

[3]Department of Chemistry, University of Colorado Boulder, Boulder, CO 80309, USA

* To whom correspondence should be addressed.

Emails: Elisa M. Miller, Elisa.Miller@nrel.gov; James L. Young, James.Young@nrel.gov

Table of Contents

Page

Table S1. Concentration Versus Time Values from Nafion 212 NH_4^+ Crossover Test at Open Circuit S-3

Table S2. Average Current and Average Full Cell Voltage Recorded During H-Cell Membrane Crossover Testing. S-3

Table S3. Properties of Membranes Tested. S-3

Table S4. Concentration Versus Time Values from PiperION-A80 NH_4^+ Crossover Test at Open Circuit S-4

Figure S1. Current (red trace) and full cell voltage (blue trace) recorded during H-cell Nafion 212 NH_4^+ crossover test with the carbon paper working electrode held at -0.5 V vs. RHE. S-5

Figure S2. NH_3 crossover signal in GDE-cell with no membrane or GDE present (blue trace) or with a single GDE present (red trace), showing ~1 min delay of crossover onset with the addition of the GDE. Prior to t = 0, N_2 was flowing through the cathode-side flow field, and at t = 0, 1.05% NH_3 in N_2 began flowing through the cathode-side flow field. S-6

Figure S3. Sample indophenol test calibration curve used for calculation of NH_4^+ concentration. The indophenol test calibration curve was re-measured for each sample set measured on a given day. S-7

Figure S4. UV-vis spectra of indophenol test solutions resulting from testing of NH_4^+ standards with concentrations 0.00 – 0.50 ppm, and UV-vis spectra of indophenol test solutions resulting from testing of graphite anode soak solutions. S-8

Discussion of Graphite Anode as a Source of Contaminating NH_3/NH_4^+ S-9

Table S1. Concentration Versus Time Values from Nafion 212 NH_4^+ Crossover Test at Open Circuit

time	*Replicate 1 cathode chamber NH_4^+ concentration*	*Replicate 1 anode chamber NH_4^+ concentration*	*Replicate 2 cathode chamber NH_4^+ concentration*	*Replicate 2 anode chamber NH_4^+ concentration*	*Replicate 3 cathode chamber NH_4^+ concentration*	*Replicate 3 anode chamber NH_4^+ concentration*
t = 0	0.510	below LOQ[a)]	0.508	below LOQ[a)]	0.501	below LOQ[a)]
t = 2 h	0.454	0.034	0.446	0.030	0.443	0.048
t = 4 h	0.429	0.068	0.408	0.079	0.398	0.098
t = 6 h	0.388	0.102	0.384	0.103	0.364	0.134

[a)] the limit of quantitation (LOQ) for the indophenol test method calibration curve was 0.007 ppm

Table S2. Average Current and Average Full Cell Voltage Recorded During H-Cell Membrane Crossover Testing.

Membrane	*Average Current i (mA)*[a)]	*Avg. $E_{cathode}$-E_{anode} (V)*[b)]
Nafion 212	-0.4 ± 0.1	-1.96 ± 0.08
Sustainion X37-50 Grade RT	-0.2 ± 0.1	-1.8 ± 0.3
Fumasep FAA 3-50	-0.7 ± 0.6	-2.01 ± 0.06
PiperION-A80	-0.4 ± 0.2	-1.97 ± 0.03
Celgard 3401	-0.4 ± 0.2	-1.9 ± 0.2

[a)]values represent average value of *i* for three replicate cells with combined standard deviation. $E_{cathode}$ = - 0.5 V vs RHE. Membrane area = 1.77 cm^2

[b)]values represent average value of $E_{cathode}$-E_{anode} for three replicate cells with combined standard deviation.

Table S3. Properties of Membranes Tested.

Membrane	NH_3/NH_4^+ released from 3x3 cm piece into 40 mL (µg) (as received)	membrane type	polymer composition	stationary charge identity	thickness (µm)	basis weight (g m^-2)
Nafion 211[1]	1.56	CEM	perfluorosulfonic acid/polytetrafluoroethylene copolymer	sulfonic acid	25	50
Nafion 212[1]	"below limit of quantitation"	CEM	perfluorosulfonic acid/polytetrafluoroethylene copolymer	sulfonic acid	50	100
Sustainion X37-50 Grade RT[2]	1.70	AEM	polystyrene/vinylbenzyl-R copolymer, R = imidazolium or pyridinium	imidazolium or pyridinium	50	(not reported)
Fumasep FAA 3-50[3]	0.95	AEM	(not reported)	(not reported)	45-55	60-85
PiperION-A80[4]	"below limit of quantitation"	AEM	poly(aryl piperidinium)	piperidinium	80	90.4
Celgard 3401[5]	1.92	porous	polypropylene	none	25	(not reported)

Table S4. Concentration Versus Time Values from PiperION-A80 NH_4^+ Crossover Test at Open Circuit

time	*Replicate 1 cathode chamber NH_4^+ concentration*	*Replicate 1 anode chamber NH_4^+ concentration*	*Replicate 2 cathode chamber NH_4^+ concentration*	*Replicate 2 anode chamber NH_4^+ concentration*	*Replicate 3 cathode chamber NH_4^+ concentration*	*Replicate 3 anode chamber NH_4^+ concentration*
t = 0	0.508	below LOQ[a)]	0.499	below LOQ[a)]	0.511	below LOQ[a)]
t = 2 h	0.514	below LOQ[a)]	0.512	below LOQ[a)]	0.508	below LOQ[a)]
t = 4 h	0.497	below LOQ[a)]	0.491	below LOQ[a)]	0.505	below LOQ[a)]
t = 6 h	0.500	below LOQ[a)]	0.510	below LOQ[a)]	0.509	below LOQ[a)]

[a)] the limit of quantitation (LOQ) for the indophenol test method calibration curve was 0.007 ppm

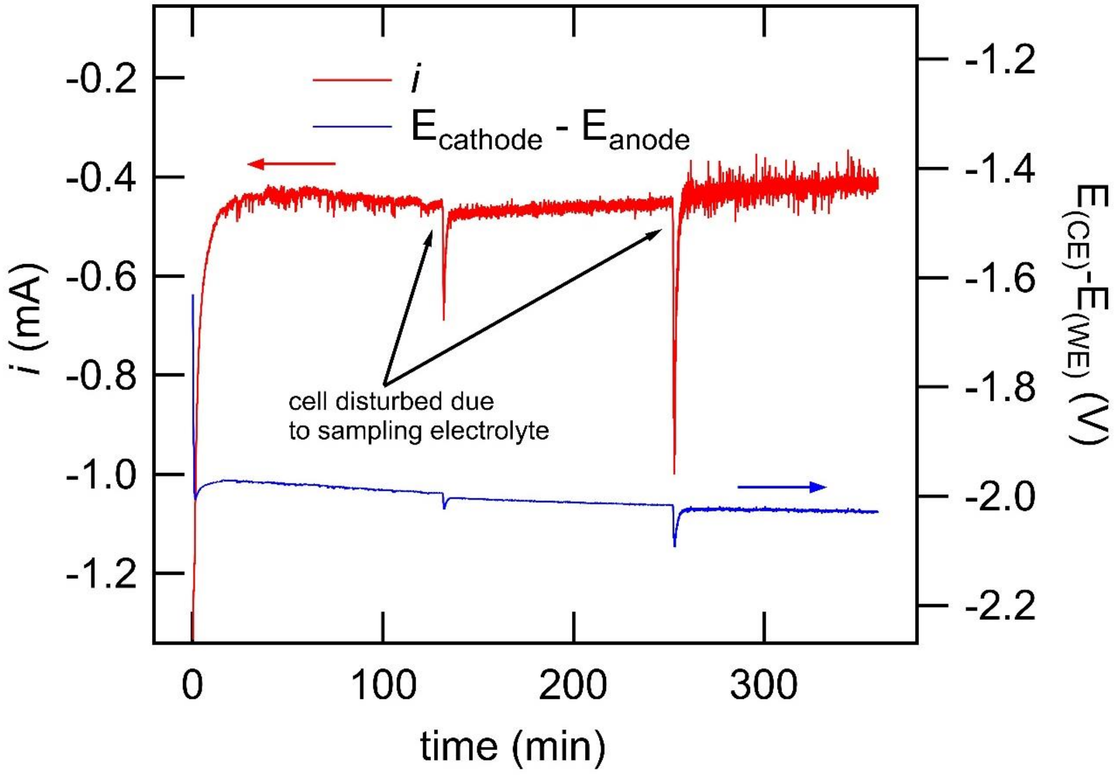

Figure S1. Current (red trace) and full cell voltage (blue trace) recorded during H-cell Nafion 212 NH_4^+ crossover test with the carbon paper working electrode held at -0.5 V vs. RHE.

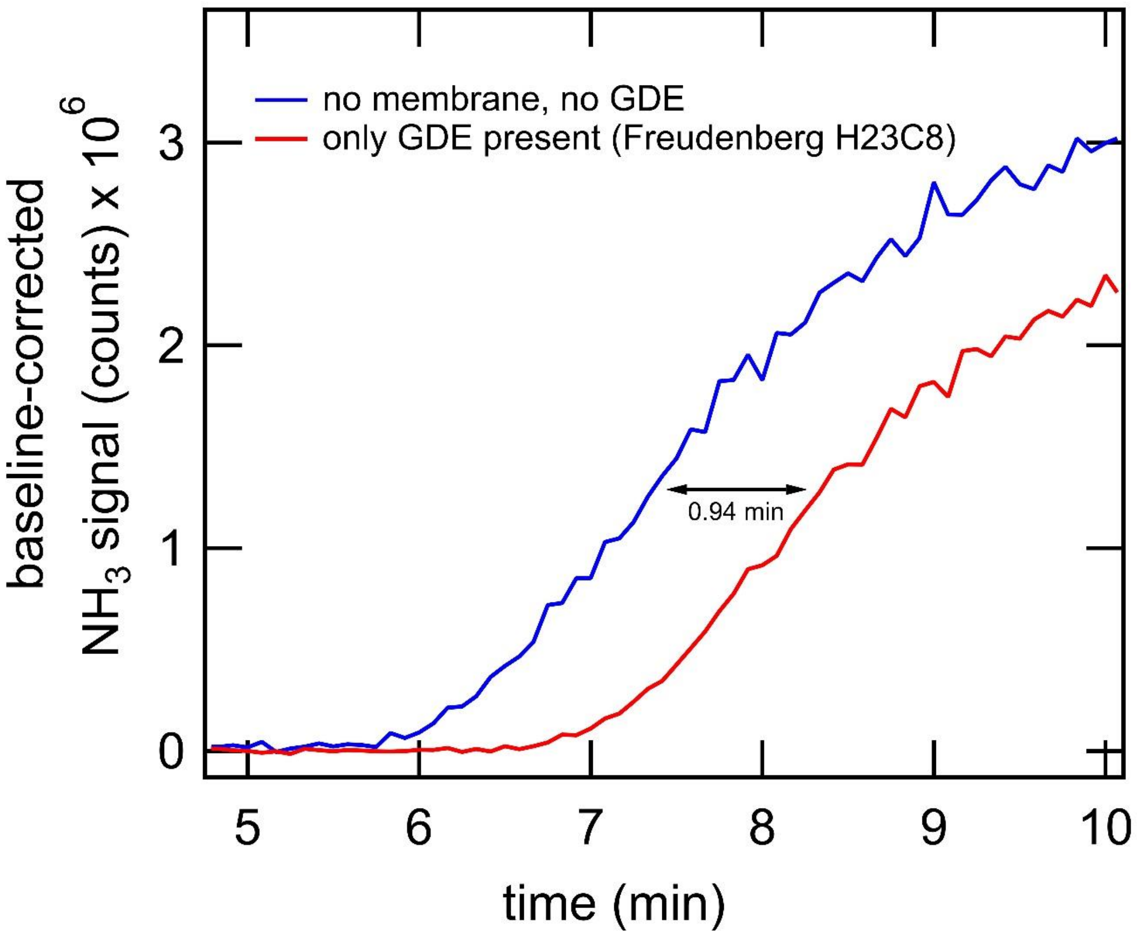

Figure S2. NH_3 crossover signal in GDE-cell with no membrane or GDE present (blue trace) or with a single GDE present (red trace), showing delay of crossover onset with the addition of the GDE. Prior to t = 0, N_2 was flowing through the cathode-side flow field, and at t = 0, 1.05% NH_3 in N_2 began flowing through the cathode-side flow field.

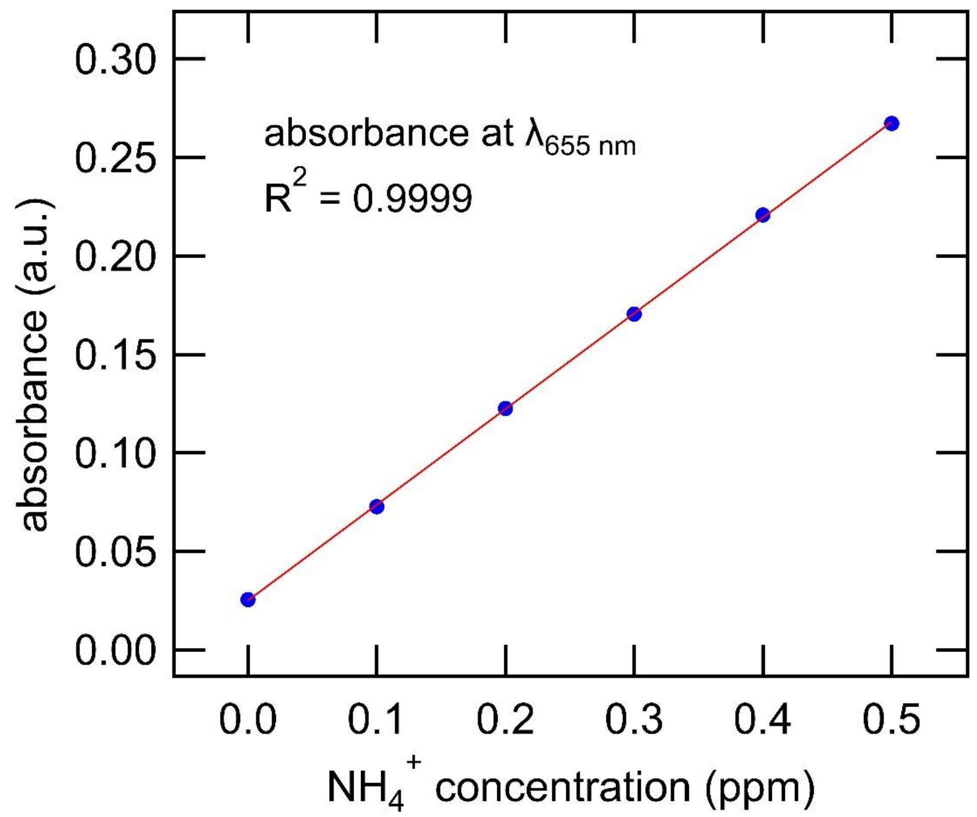

Figure S3. Sample indophenol test calibration curve used for calculation of NH_4^+ concentration. The indophenol test calibration curve was re-measured for each sample set measured on a given day.

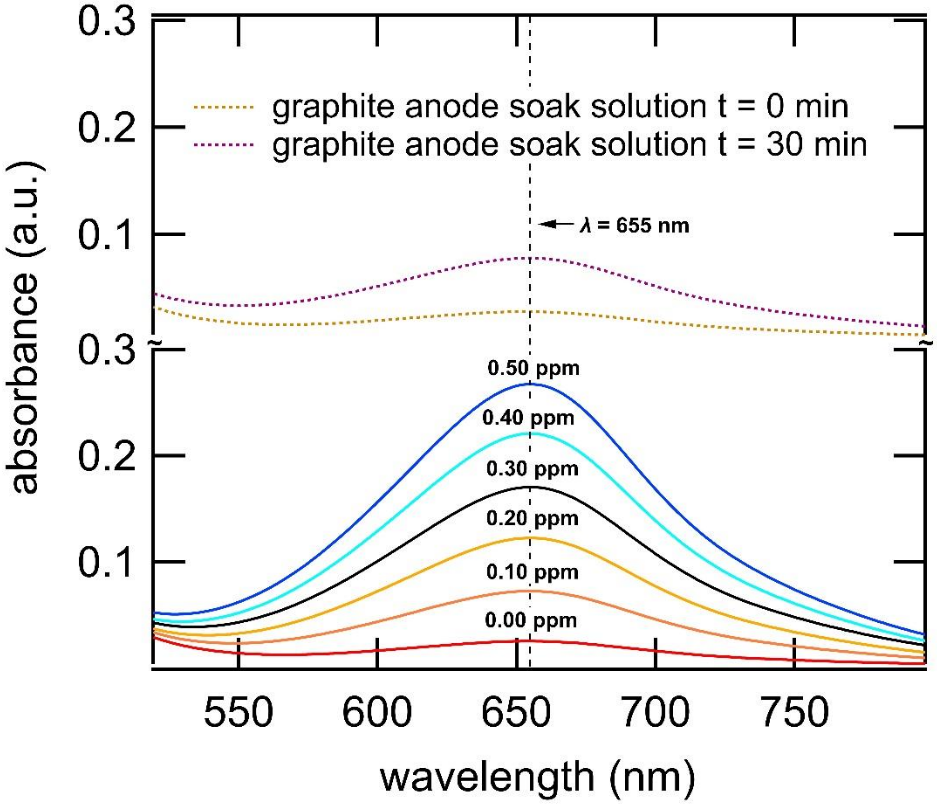

Figure S4. UV-vis spectra of indophenol test solutions resulting from testing of NH_4^+ standards with concentrations 0.00 – 0.50 ppm, and UV-vis spectra of indophenol test solutions resulting from testing of graphite anode soak solutions.

Discussion of Graphite Anode as a Source of Contaminating NH_3/NH_4^+

In initial H-cell experiments, an anomalously high NH_3/NH_4^+ concentration (in the range of 0.05 – 0.10 ppm) was sometimes measured in the anode-chamber of the H-cell at t = 0. In the experimental procedure, the t = 0 aliquot is removed from the fully assembled H-cell, and so it is possible that the increased t = 0 concentration in the anode-chamber is the result of release of NH_3/NH_4^+ from the graphite anode (the counter electrode). Here, a control experiment is conducted to evaluate the graphite anode as a source of NH_3/NH_4^+ contamination. In this experiment, three graphite anodes are stored in a container which was open to laboratory air for 10 days, and following this, the anodes are rinsed with deionized (DI) water for 5 s and then placed in 0.1 M HCl (15 mL) for 30 min. The graphite anodes release an average of 1.7 ± 0.4 μg NH_3/NH_4^+ into the soak solution, which represents an unacceptably high level of contamination. The original source of NH_3/NH_4^+ within the graphite anodes is unknown, but it may represent absorption of atmospheric NH_3 on a timescale of days.

To correct the apparent NH_3/NH_4^+ contamination of graphite anodes, the graphite anodes are cleaned electrochemically via electrooxidation for retention experiments. In the procedure, cyclic voltammetry is used to oxidize NH_3/NH_4^+ trapped within the graphite anodes. The anodes are cycled between 0.00 and 2.00 V vs. Ag/AgCl 20 times, with a scan rate of 500 mV/s. In tests of the electrochemically cleaned anodes, only one out of the three graphite anodes tested shows a measurable quantity of NH_4^+ (0.4 μg) released into the 0.1 M HCl (15 mL) test solution. In all electrochemical H-cell NH_3/NH_4^+ crossover experiments reported here, the graphite anodes are cleaned by cyclic voltammetry prior to the experiment, and as a further step, chronoamperometry is then used to apply -0.5 V vs. Ag/AgCl to the working electrode in the fully assembled H-cell, which holds the graphite anode at an oxidizing potential, for 1 h (followed by copious rinsing of

the cell with fresh electrolyte). In the design of NH_3-generating electrolyzers for catalyst testing, care must be taken to eliminate sources of contaminating NH_3/NH_4^+ from all components of the electrochemical cell, which may include the electrodes.

To ensure the measured NH_3/NH_4^+ released by the graphite anodes is not the result of an error in the indophenol test UV-vis assay, UV-vis spectra of the indophenol test calibration curve are compared with UV-vis spectra of the graphite anode soak solutions (t = 0 and t = 30 min, Figure S3). The spectra of the tested solutions are qualitatively similar, with the calibration curve solutions and the t = 30 min sample solution both showing an absorption maximum at 655 nm, which arises from the presence of indophenol.

References

(1) Peron, J.; Mani, A.; Zhao, X.; Edwards, D.; Adachi, M.; Soboleva, T.; Shi, Z.; Xie, Z.; Navessin, T.; Holdcroft, S. Properties of Nafion® NR-211 membranes for PEMFCs. *Journal of Membrane Science* **2010,** *356*, 44-51.

(2) Li, W.; Fang, J.; Lv, M.; Chen, C.; Chi, X.; Yang, Y.; Zhang, Y. Novel anion exchange membranes based on polymerizable imidazolium salt for alkaline fuel cell applications. *Journal of Materials Chemistry* **2011,** *21*, 11340-11346.

(3) Technical Data Sheet - fumasep® FAA-3-50. https://www.fuelcellstore.com/spec- sheets/fumasep- faa- 3-50-technical-specifications.pdf (accessed 03-16-2023).

(4) PiperION Anion Exchange Membrane, 80 microns, Self-Supporting. https://www.fuelcellstore.com/versogen-piperion-aem-self-supporting-80um-73800002 (accessed 03- 16-2023).

(5) Celgard® 3401 Monolayer Microporous Membrane. https://www.celgard.com/storage/components/3401_com_october2021_20020.pdf (accessed 03- 16- 2023).

(6) Polybenzimidazole (PBI) Film. https://pbipolymer.com/wp- content/uploads/2016/05/Film_Brochure.pdf (accessed 03-16-2023).
